# Proximal tibiofibular joint dislocation is rare in knee dislocations type Schenck III or higher

**DOI:** 10.1002/jeo2.70556

**Published:** 2025-11-18

**Authors:** Ben Louis Wagener, Timo Stausberg, Bertil Bouillon, Thomas Rudolf Pfeiffer, Thomas Stein, Daniel Guenther

**Affiliations:** ^1^ Department of Orthopaedic Surgery, Trauma Surgery, and Sports Medicine, Cologne Merheim Medical Center Witten/Herdecke University Witten Germany; ^2^ Department of Arthroscopic Surgery Sports Traumatology and Sports Medicine, BG Klinikum Duisburg gGmbH Duisburg Germany; ^3^ SPORTHOLOGICUM Frankfurt Frankfurt Am Main Germany; ^4^ Department of Sports Medicine Goethe University Frankfurt Frankfurt Am Main Germany

**Keywords:** computed tomography, dislocation, knee, proximal tibiofibular joint, PTFJ

## Abstract

**Purpose:**

The purpose of the study was to determine the prevalence of proximal tibiofibular joint (PTFJ) dislocations in knee dislocations classified as Schenck type ≥III and to compare with uninjured contralateral knees, which served as control cohort.

**Methods:**

Patients treated at Cologne Merheim Medical Center with knee dislocation ≥III between 2015 and 2022 were included, while one control group consisted of uninjured contralateral knees. In addition to established PTFJ‐specific parameters, two novel computed tomography‐based PTFJ parameters were implemented on scans obtained a mean of 5 days post‐trauma. The fibula lateralisation quantifies the lateral shift of the proximal fibula joint line in relation to the tibial PTFJ joint line. The posterior fibula area quantifies the fibula position in the sagittal plane with reference to the posterior tibial margin.

**Results:**

A total of 107 knee joints were included, comprising 40 with knee dislocation ≥III, 24 uninjured contralateral knees and 43 chronically isolated anterior cruciate ligament insufficient knees. The overall cohort had a median posterior fibula area of 92.7% and a fibula lateralisation of 0.0%. Complete PTFJ dislocation was observed in 5.4% of knee dislocation cases, all in type IV injuries. Fibula lateralisation analysis indicated PTFJ subluxation in an additional 21.6% of cases. No PTFJ dislocations were present in the contralateral knee group. The knee dislocation group differed significantly from the matched contralateral knee group with regard to fibula lateralisation (*r* = 0.43; *p* = 0.007), whereas no differences were observed for inclination‐horizontal (*p* = 0.620), inclination‐fibular axis (*p* = 0.082) and obliquity (*p* = 0.602).

**Conclusions:**

This study demonstrates a 5.4% prevalence of PTFJ dislocation in knee dislocations ≥III, which is lower than previously reported. The incidence of PTFJ dislocation in knee dislocations type IV is high at 33.3%. Fibula lateralisation and posterior fibula area are suitable parameters for assessing anterolateral dislocation of the proximal fibula. Inclination‐horizontal, inclination‐fibular axis and obliquity show no relevant side‐to‐side differences in individuals.

**Level of Evidence:**

Level III.

**Abbreviations**
APTFL: anterior proximal tibiofibular ligament
CT: computed tomography
FL: fibula lateralisation
KD: knee dislocation
PCL: posterior cruciate ligament
PLC: posterolateral corner
PPTFL: posterior proximal tibiofibular ligament
PTFJ: proximal tibiofibular joint

## INTRODUCTION

The dislocation of the proximal fibula was first described by Nélaton in 1868 [25]. Nevertheless, the proximal tibiofibular joint (PTFJ) has since rarely been the subject of scientific studies [9, 32] and PTFJ dislocations are even considered underinvestigated or understudied injuries [14, 22]. Through foundational works on PTFJ anatomy [3] and Ogden's publications [28, 29, 30], which systematically addressed PTFJ dislocations and subluxations beyond small case series, the PTFJ has gained increasing attention.

The fibular articular surface is oriented anteriorly, medially, and proximally to interact with the tibia [9]. Based on its joint inclination angle in the sagittal plane, PTFJs can be classified into a horizontal type, with an inclination of less than 20°, and an oblique type, with an inclination greater than 20° [29]. The oblique joint type is significantly more prevalent overall [4, 29] and accounts for approximately 70% of PTFJ dislocations [27]. The capsuloligamentous structures of the PTFJ, similar to other components of the posterolateral corner (PLC) [43], demonstrate a high degree of anatomical variability. The PTFJ consists of the anterior proximal tibiofibular ligament (APTFL) and the posterior proximal tibiofibular ligament (PPTFL), both with a medioproximal to laterodistal fibre orientation. These ligaments can further be differentiated in up to four individual bundles of the APTFL and three of the PPTFL [2]. Numerous other studies, however, assign fewer bundles to both ligamentous complexes [9, 30, 31, 36]. The APTFL complex is thicker [2, 8, 9] and demonstrates higher mechanical resistance [23] compared to the posterior ligamentous complex.

PTFJ dislocations can be classified as idiopathic or traumatic and, in descending order of incidence, are categorised into anterolateral, posteromedial, proximal and distal dislocations [12, 13, 27]. Anterolateral dislocation has a prevalence of at least 77% and is by far the most common type of PTFJ dislocation [13, 18, 21, 26, 28]. Little is known about PTFJ subluxation and precise diagnostic criteria remain undefined [28, 32].

Clinical examination including side‐to‐side comparison plays a crucial role in detecting PTFJ instability, Ballottement [33, 37] and Sijbrandij test [38] are commonly used manoeuvres. In simple cases, diagnosing proximal fibula dislocation based on anterior‐posterior (a.p.) and lateral radiographs is possible; additional support can be provided by roentgenologic criteria [14]. However, Keogh et al. [17] demonstrated that detection accuracy is only 72.5% in a cohort including healthy joints. Additional radiographs of the contralateral knee can improve accuracy. The highest diagnostic accuracy can be achieved when computed tomography (CT) imaging is incorporated; nevertheless, the sensitivity for anterolateral PTFJ dislocations remained at only 71%. Computed tomography is still the diagnostic tool of choice in unclear cases [17, 42]. The authors hypothesised that the positioning of the fibula in a.p. orientation in axial CT scans, referenced to the posterior tibial margin, could be indicative of PTFJ dislocation [17].

To date, only two case series and a few case reports [10, 12, 39] have examined the coincidence of knee joint and PTFJ dislocations. One case series focused on acute knee dislocations (KD) [16], while the other investigated chronic multiligamentous knee instability involving at least two ligaments [33], assessing the presence of PTFJ instability. PTFJ instability was identified in 9% of acute and 10.7% of chronic KDs, a total of 21 cases, based on preoperative or intraoperative clinical examination. PTFJ dislocations are often overlooked not only as isolated injuries [30] but also in multiligamentous knee injuries [18].

The purpose of the study was to determine the prevalence of PTFJ dislocation in KDs classified as Schenck type ≥III and to compare with isolated anterior cruciate ligament (ACL) insufficiency and contralateral knees. These knees serve as an appropriate control cohort, as they are not associated with PTFJ injuries and routinely undergo CT imaging in preparation for surgery. The hypothesis was that PTFJ dislocations appear to be more prevalent in KDs ≥ III than previously reported, while exhibiting low prevalence in chronically ACL‐insufficient and uninjured contralateral knees.

## METHODS

Computed tomography imaging data of 107 knee joints from the Cologne Merheim Medical Center, collected between 2015 and 2022, matched inclusion criteria and were retrospectively analysed. The sample consisted of three subgroups: 40 patients (29 male) with KDs classified as Schenck ≥ III [24, 34] formed the knee dislocation cohort (LUX). In most cases, a spiral CT was performed as part of the initial trauma assessment, while a minority underwent dedicated knee scans for preoperative planning, resulting in a mean imaging interval of 4.97 days post‐trauma. Bilateral imaging was performed in 24 of these patients (16 male), allowing the formation of a cohort with uninjured contralateral knees (CL). Inclusion criteria were age ≥16 years, acute KD classified as Schenck III–V, meeting the CT morphological criteria listed below, no further ligament reconstruction and no further knee operation. The third group included 43 patients (30 males) with first failure of ACL reconstruction – following a description as chronic monoligamentous ACL insufficiency – who routinely underwent unilateral CT imaging as part of preoperative planning for bone grafting of the ACL tunnels. Inclusion criteria were age ≥16 years, single previous ACL reconstruction, meeting the CT morphological criteria listed below, no further ligament reconstruction and no further knee operation. Exclusion criteria for all groups included age <16 years, previous knee surgeries or ligament injuries, rheumatic diseases and tumours affecting the examined extremity.

**Figure 1:**
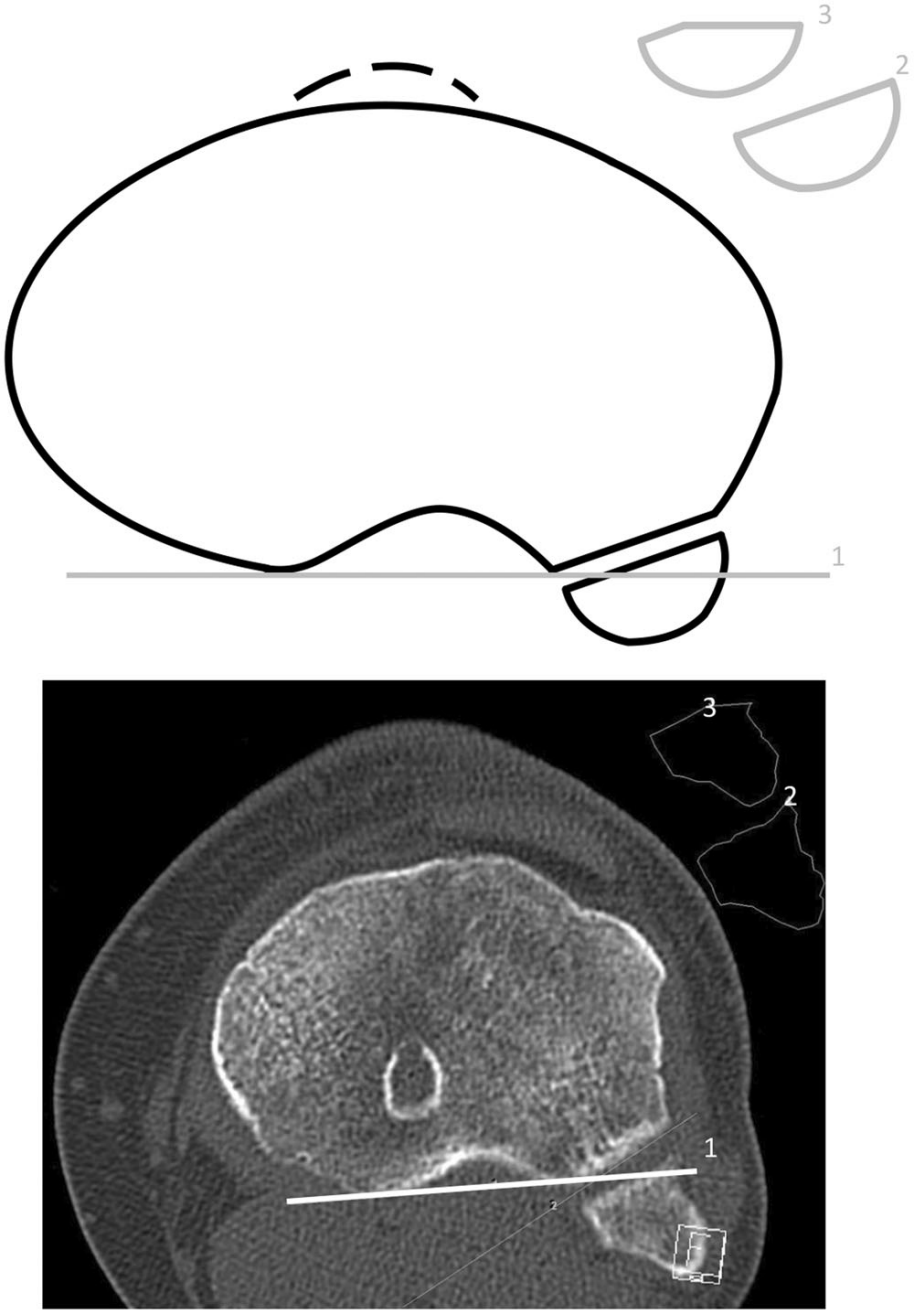
Posterior fibula area (PFA) of the proximal tibiofibular joint. *Note*: axial plane of an exemplary left knee as a schematic illustration (top) and computed tomography (bottom), not included in the cohort. (1) tangent to the posterior tibial margin, (2) total fibula area (min. 150 mm^2^), (3) fibula area posterior to the tibial margin. (1) divides the axial fibula area into a posterior (3) and an anterior part. (2) and (3) can be used to calculate the PFA.

In addition to demographic data (age, height, weight, body mass index), the following parameters were recorded in trauma CT: PTFJ joint type [29], PTFJ inclination‐horizontal [44], PTFJ inclination‐fibular axis [4, 8, 9, 44], PTFJ obliquity [41], posterior fibula area, fibula lateralisation and fibula fractures. In inclination measurements, the anterior tilt of the PTFJ in the sagittal plane was referenced against a horizontal line (PTFJ inclination‐horizontal) and the axis of the proximal fibula (PTFJ inclination‐fibular axis). In axial images, the tangent along the posterior tibial margin, combined with the extended PTFJ joint line defined the PTFJ obliquity. This tangent was also used in the subsequent posterior fibula area calculation (Figure 1). Clinical follow‐up data were available from 27 patients at a mean interval of 17 months following trauma to evaluate postoperative knee stability, including assessment by varus stress testing. Lateral knee instability was defined as grade II (double positive) or higher finding in the varus stress test. A follow‐up examination is missing in 13 patients for no known reason. Complications were defined as wound healing disorders, infections, compartment syndrome, post‐traumatic joint stiffness, fibular pseudarthrosis and the need for revision surgery. Injury events were categorised as ultra‐low‐velocity events (daily activities by foot), low‐velocity events (sports, non‐motorised vehicles, <30 km/h) and high‐velocity events (motorised vehicles, >30 km/h).

Image analysis was performed using Centricity PACS 4.0 (Radiologist Workstation, GE Healthcare, Barrington, USA) by two experienced medical examiners from the Department of Orthopedic and Trauma Surgery. Before the study, both examiners were prepared using a methodological description of the measurements to be performed. Each examiner performed two independent measurements with an interval of minimum 4 weeks. In the second measurement round, 40 randomly selected knee joints from the knee dislocation, contralateral knee and ACL‐insufficiency cohorts were analysed and measured by the same rater again to calculate the intra‐rater reliability.

Using multiplanar localisation in the facultative imaging plane, the centre of the PTFJ was identified for the mandatory plane. As a control mechanism, all images of the mandatory plane that provided a representative cross‐section of the PTFJ were counted. The selected image for measurement corresponded to the median of the total number of PTFJ imaging planes. The following criteria further defined the appropriate imaging plane: the typical anatomical shape and positioning of the fibular and tibial head were visible, the PTFJ joint line was clearly delineated, the articulating joint surfaces were parallel to each other, the articulating joint surfaces were as big as possible, maintaining the largest possible joint‐specific distance, and the apex fibulae was not recognised as joint surface.

### Posterior fibula area

The availability of the axial plane is mandatory. The fibular surface must be sufficiently large to ensure that the joint surface rather than the apex of the fibula is represented (minimum area: 150 mm²; see (2) in Figure 1).

The contour of the entire fibular head is defined by using at least 30 cortical markings along its circumference, and the total fibular area is calculated (see (2) in Figure 1). This measurement is repeated with at least 25 markings, excluding the anterior fibular surface anterior to the posterior tibial margin (1), to determine the posterior portion of the fibula area (3). The posterior fibula area is then calculated according to Formula 1. If the posterior tibial margin line (1) is positioned anterior to the fibula, the posterior fibula area corresponds to the total fibular area and is therefore 100%.

(1) $$\text{Posterior fibula area},\;\text{PFA}\;[\%]=\left(\frac{\left[3\right]}{\left[2\right]}\right)\times 100.$$

### Fibula lateralisation

**Figure 2:**
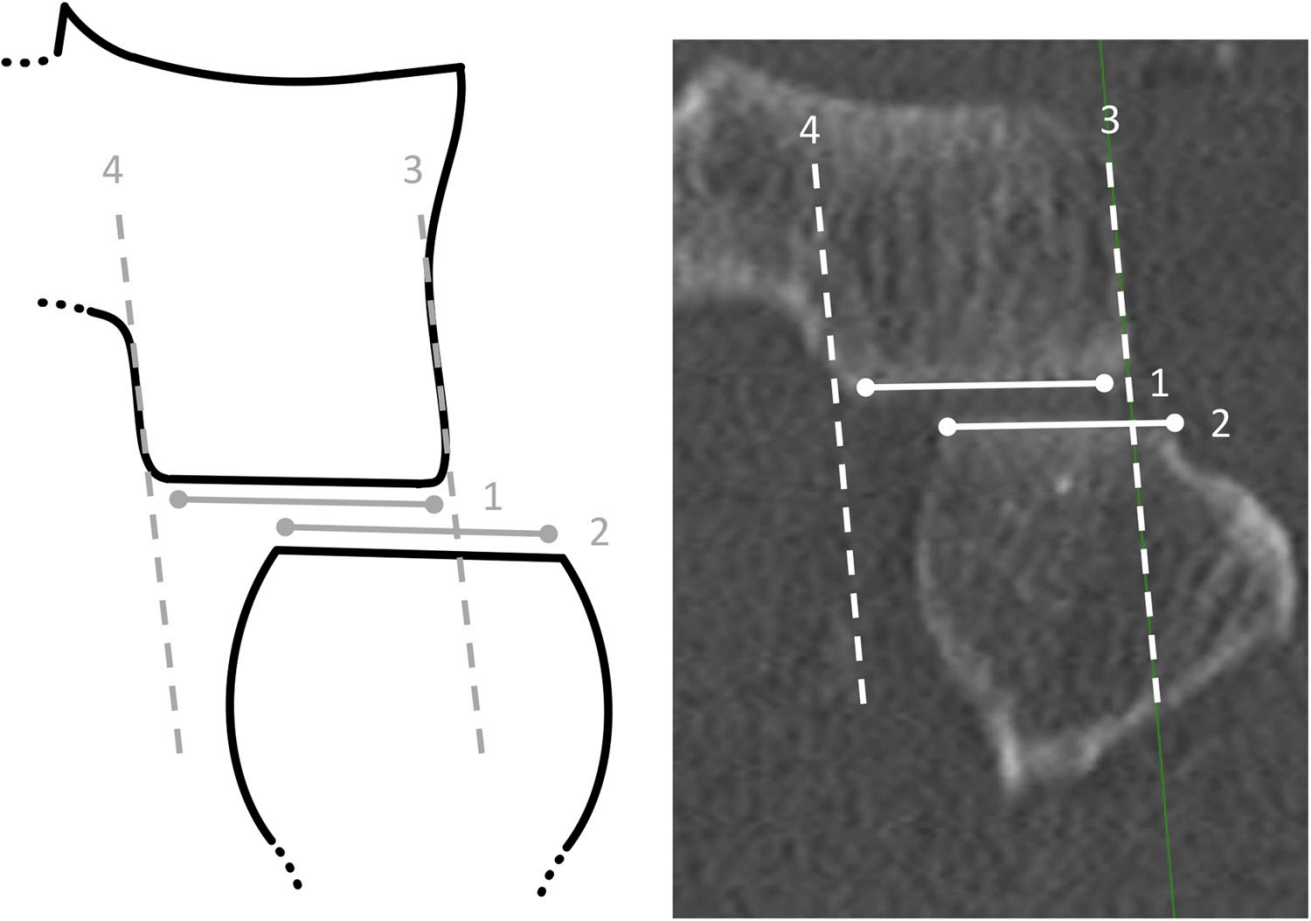
Visualisation of fibula lateralisation (FL) in the proximal tibiofibular joint (PTFJ). *Note*: coronar plane of a left PTFJ as a schematic illustration (left) and computed tomography (right), knee dislocation group. (1) tibial PTFJ joint surface, (2) fibular PTFJ joint surface, (3) tangent of the distal lateral tibia, (4) mirrored tangent of the lateral tibia. By employing the lateral tibia tangent (3), the lateral displacement of (2) relative to (1) can be precisely visualised.

The availability of the coronal plane is mandatory. The selected plane additionally illustrates the femur. The tibial (1) and fibular (2) joint surfaces are parallel to each other and have a similar length (Figure 2) [15].

The tangent of the distal lateral tibia cortex (3) is mirrored to the medial boundary of the tibial PTFJ joint surface (4). Using the two tibial joint surface reference lines (3) and (4), a lateral or medial overlap of the fibula could be clearly identified. Lateralisation is present if the medial and lateral boundaries of the fibular joint surface simultaneously exceeded the tibial joint surface reference lines (3) and (4) in the same lateral direction. In contrast, physiological joint conditions are present if only one of the fibular joint surface boundaries exceeds the corresponding tibial one.

**Figure 3:**
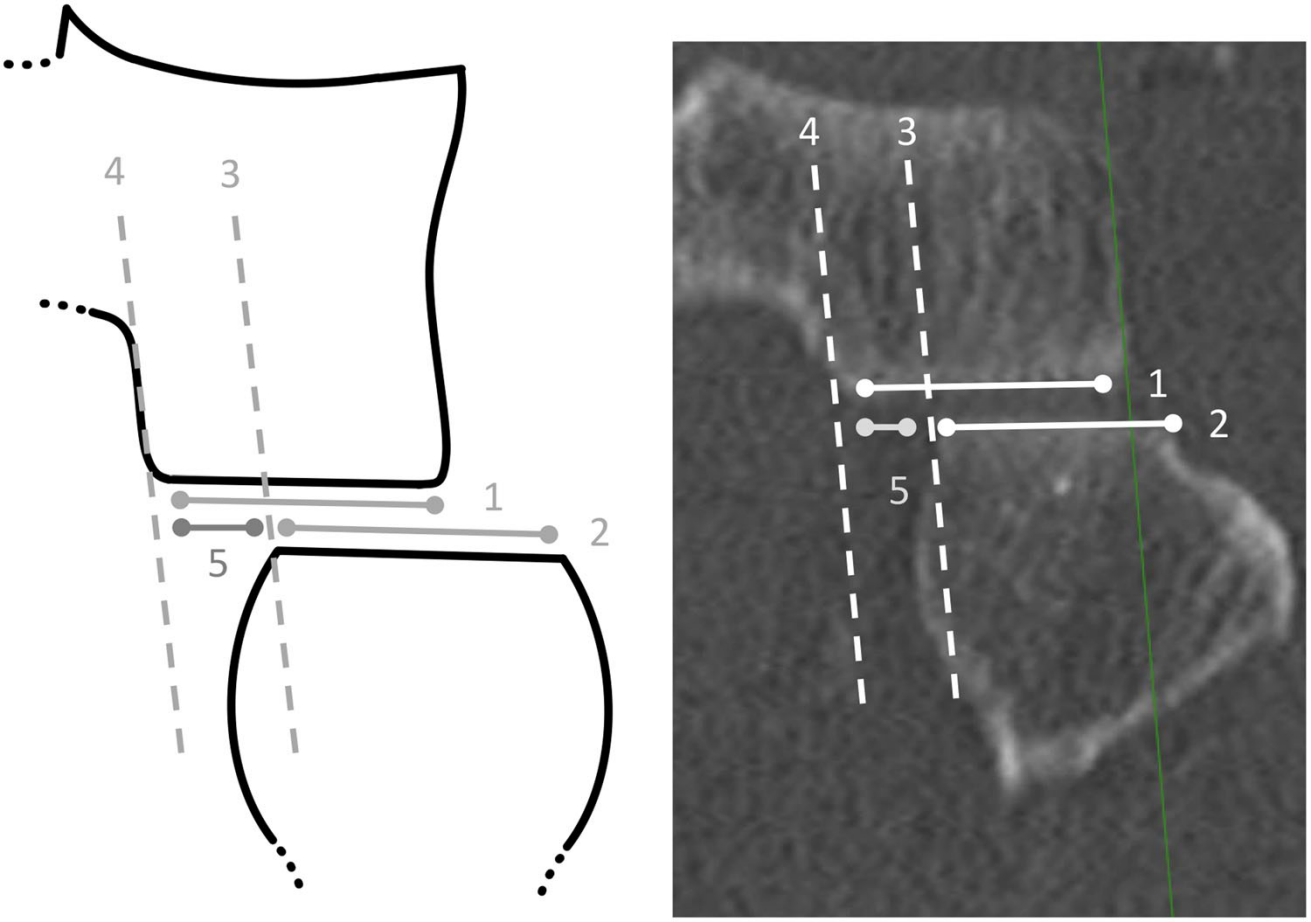
Measurement of fibula lateralisation (FL) in the proximal tibiofibular joint (PTFJ). *Note*: coronar plane of a left PTFJ as schematic illustration (left) and computed tomography (right), knee dislocation group. (1) tibial PTFJ joint surface, (2) fibular PTFJ joint surface, (3) shifted tangent of the distal lateral tibia, (4) mirrored tangent of the lateral tibia, (5) distance of the medial free PTFJ joint surface of the tibia. The ratio of (5) to (1) is calculated to quantify the lateral displacement of the fibular PTFJ joint surface (2) relative to the tibial PTFJ joint surface (1).

If a lateralisation is present, the lateral tibial joint surface reference line (3) is shifted to the medial boundary of the fibular joint surface, and the distance (5) between (3) and (4), aligned parallel to the PTFJ joint line, is determined (Figure 3).

Distance (5) describes the lateral translation of the fibula in the frontal plane relative to the articulating tibial joint surface. Fibula lateralisation (Formula 2) defines the extent to which the fibular PTFJ joint surface is laterally displaced relative to the tibial PTFJ joint surface.

(2) $$\text{Fibula Lateralisation},\;\text{FL}\;[\%]=\left(\frac{\left[5\right]}{\left[1\right]}\right)\times 100.$$

In this study the integrity versus dislocation of the PTFJ is determined exclusively by preoperative CT data. Joints with a fibula lateralisation > 27.7% are classified as injured PTFJs, using the upper limit of the 95% confidence interval (CI) of the knee dislocation cohort as the cut‐off value. Proximal tibiofibular joints that are assessed as dislocated due to apparent malposition of the proximal fibula in CT and simultaneously exhibited a fibula lateralisation > 27.7% were classified as complete dislocation. Proximal tibiofibular joints that were assessed as not dislocated due to absence of malposition but nevertheless exhibited a fibula lateralisation > 27.7% were classified as subluxation.

### Statistics

Statistical analysis was performed using IBM SPSS Statistics (2021, Version 28.0.0.0 (190), Chicago, USA). A a‐priori power analysis was not conducted due to the lack of comparable publications. A value of *p* < 0.05 was considered statistically significant. Normal distribution was assessed using the Kolmogorov–Smirnov and Shapiro–Wilk tests; non‐normally distributed data were reported using the median, minimum, maximum, and interquartile range (median [minimum–maximum; interquartile range]).

Group comparisons for statistically significant differences in metric data were performed using the Mann–Whitney *U* test. To assess relationships between nominal or ordinal variables, the chi‐square test and Fisher's exact test were applied. Effect size was evaluated using Cramer's V. An effect size of *r* < 0.1 was considered as weak, 0.1–0.3 as moderate, and > 0.5 as strong [7]. The Spearman correlation was applied to examine associations between metric or ordinal, non‐normally distributed variables.

Inter‐ and intra‐class correlation coefficients (ICC) were used to assess inter‐ and intra‐observer reliability. The ICC was reported with the 95% CI and *p*‐value and was classified as poor (<0.4), moderate (0.4–0.59), good (0.6–0.74) or excellent (≥0.75) [6].

## RESULTS

The total cohort showed a median posterior fibula area of 92.7% [18.5–100.0; 19.8] and a fibula lateralisation of 0.0% [−13.5 to 150.5; 8.1]. PTFJ obliquity correlated negatively with the posterior fibula area (*r*_s_ = ‐0.80; *p* < 0.001). A total of two complete PTFJ dislocations (2.0%) and eight PTFJ subluxations (8.2%) were detected in CT, all belonged to the knee dislocation cohort. Two cases of fibular medialization were observed. Knee joints with PTFJ dislocation or subluxation differed significantly from all other knee joints in terms of fibula lateralisation (*r* = 0.63; *p* < 0.001). 13/15 fibular fractures were assigned to the knee dislocation cohort. Six cases of avulsion fractures and two of multifragmentary fractures where detected. Fibular fractures were exclusively found in oblique‐type PTFJs.

### Knee dislocation (LUX) cohort

The upper limit of 95% CI for fibula lateralisation was 27.7%. The median posterior fibula area was 88.5% in PTFJ dislocated or subluxated joints and 95.7% in other KDs classified as Schenck ≥ III; however, this difference was not significant (*p* = 0.451) in contrast to the difference observed for fibula lateralisation (*r* = 0.81, *p* < 0.001). A total of 16 Schenck IIIL injuries (40.0%), 13 Schenck IIIM injuries (32.5%), 7 Schenck IV injuries (17.5%) and 4 Schenck V injuries (10.0%) were recorded.

**Figure 4:**
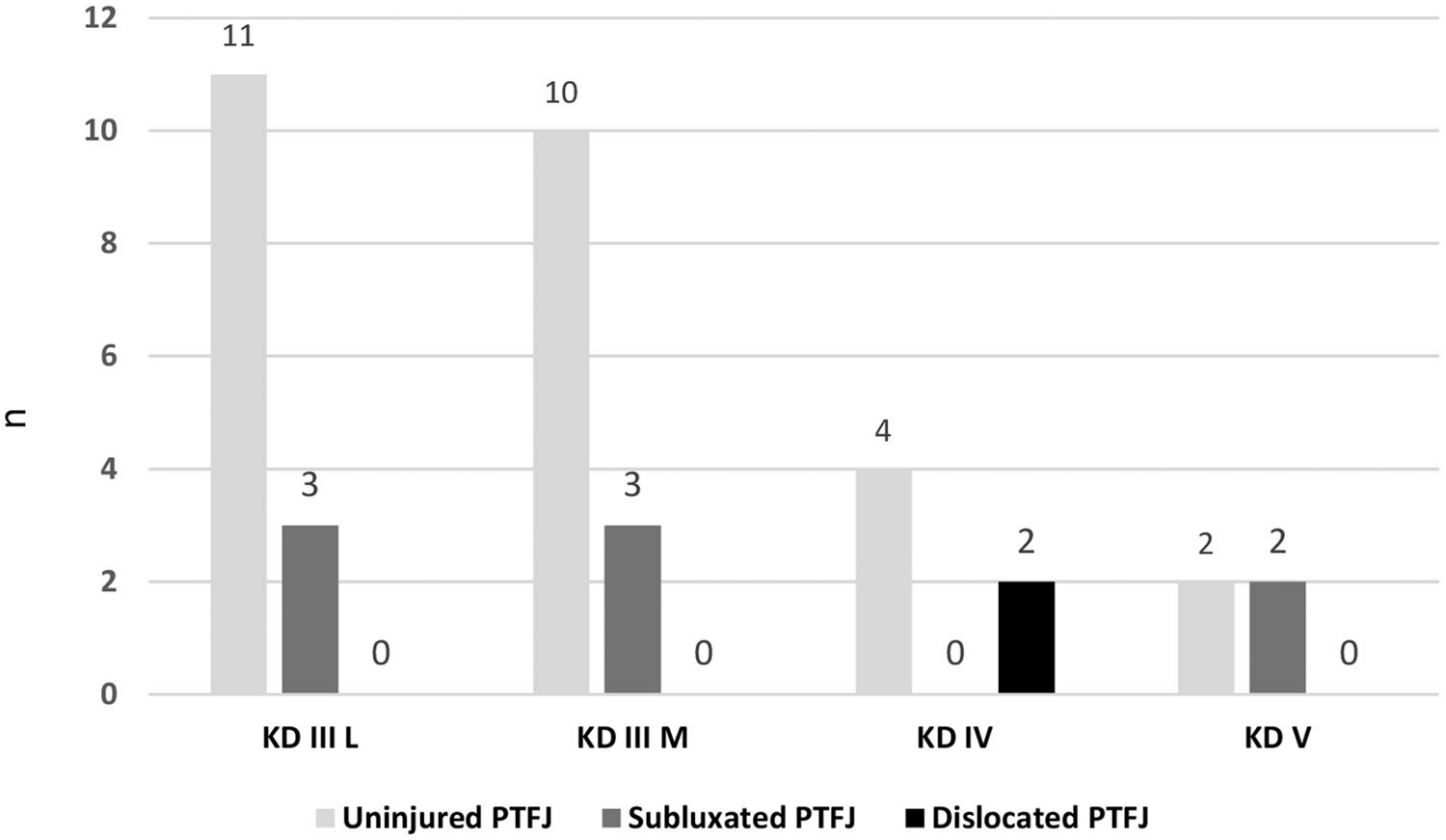
Proximal tibiofibular joint dislocation or subluxation in knee dislocations (KD) ≥ III Schenck.

The trauma mechanisms included five unknown injury mechanisms, ultra‐low‐velocity (28.6%), low‐velocity (37.1%) and high‐velocity traumata (34.3%). A total of 5.4% (2/37) of PTFJs showed complete dislocation, while 21.6% (8/37) showed subluxation (Table 1). Both cases of PTFJ dislocation occurred in KDIV cases (2/6), resulting in an incidence of 33.3% in KDs type Schenck IV and an association between the Schenck types and complete PTFJ dislocation (Cramer's *V* = 0.498; *p* = 0.035). Knee dislocation IIIL demonstrated no complete PTFJ dislocation. In contrast, PTFJ subluxations showed no association with Schenck types (*p* = 0.247), they were found in KD IIIM (3/8, 37.5%), KD IIIL (3/8, 37.5%) and KD V cases (2/8, 25.0%, Figure 4). All two dislocated and eight subluxated PTFJs were oblique type PTFJs with 20% resulting from a high‐velocity mechanism and 80% caused by low‐ or ultra‐low‐velocity trauma. Eight out of 10 PTFJ dislocation or subluxation cases showed involvement of the (postero‐)lateral ligament complex. Fibula lateralisation and therefore PTFJ dislocation status could not be estimated in 3/40 KD cases due to missing CT data (2× KD IIIL and 1× KD IV).

**Table 1:** Patients with dislocation or subluxation of the proximal tibiofibular joint (PTFJ) in knee dislocations ≥ III Schenck.

							PTFJ			
No.	Sex	Age	Side	Trauma	Class.	Ligament‐Rupture	Type	FL	Larson	Lat.
1	m	38	L	2	4 N	ACL, PCL, LCL, MCL	O	150.5	No	‐
2	m	37	R	3	4	ACL, PCL, LCL, MCL	O	101.0	Yes	++
3	w	85	L	1	5	ACL, PCL, LCL, MCL	O	49.4	No	‐
4	w	53	R	2	3 M	ACL, PCL, MCL	O	36.7	n.a.	‐
5	m	42	L	2	3 M	ACL, PCL, MCL	O	36.3	n.a.	‐
6	m	62	R	1	5 N	ACL, PCL, LCL, Pop, Bic	O	36.0	No	‐
7	m	58	L	3	3 L	ACL, PCL, LCL, Bic	O	35.8	Yes	++
8	w	21	L	2	3 M	ACL, PCL, MCL, Bic	O	32.8	No	‐
9	w	62	L	1	3LN	ACL, PCL, LCL, Bic	O	32.6	Yes	n.a.
10	w	60	R	1	3LNC	ACL, PCL, LCL, Pop	O	32.1	No	++

*Note*: 1 = ultra‐low‐velocity, 2 = low‐velocity, 3 = high‐velocity, ‐ = negative, ++ = double positive

Abbreviations: PTFJ = proximal tibiofibular joint, m = male, w = female, L = left, R = right, Class. = Schenck classification, FL = fibula lateralisation, Lat. = lateral instability in varus stress test, ACL = anterior cruciate ligament, PCL = posterior cruciate ligament, MCL = medial collateral ligament, LCL = lateral collateral ligament, Bic = biceps tendon, Pop = popliteus tendon, O = oblique, n.a. = not available

Table 1 illustrates the cases of KDs with complete PTFJ dislocation (patients nos. 1–2) and PTFJ subluxation (patients nos. 3–10). Patient no. 1 underwent ligament bracing of the ACL, posterior cruciate ligament (PCL) and medial collateral ligament repair with ligament brace, cerclage of the fibular head, anterolateral meniscus root refixation, suturing of lateral meniscus middle body and neurolysis of the peroneal nerve 5 days post‐trauma. At 6 months postoperatively, clinical evaluation revealed a stable knee joint with fibular head pseudarthrosis and persisting foot drop since trauma. Patient no. 2 underwent PCL reconstruction, lateral reconstruction according to Larson [19] and medial reconstruction according to Lind et al. [20] after a femoral shaft fracture and wound healing disorder following femoral nailing. At 20 months postoperatively, both the varus stress test and the posterior drawer test remained positive.

The analysis of treatment data showed, that in no case of 40 KDs a PTFJ dislocation or subluxation was named, and consequently, no targeted therapy was documented.

Post‐traumatic sensory impairments were present in four cases, and motor impairments in six cases. Postoperative complications were documented in 9 of 40 patients (22.5%), comprising arthrofibrosis (*n* = 2), septic arthritis (*n* = 1), superficial soft‐tissue infection (*n *= 2), impaired wound healing (*n* = 1), acute compartment syndrome (*n* = 1), pseudarthrosis (*n* = 1), graft‐fixation failure (*n* = 1) and bypass‐graft occlusion (*n* = 1). Five of these nine patients showed PTFJ dislocation or subluxation.

The knee dislocation cohort (*n* = 40) can be stratified into cases with (*n* = 29) and without (*n* = 11) injury to the (postero‐) lateral ligament complex. The subgroup without lateral involvement consisted exclusively of KD IIIM injuries (11/11, 100%). Within the subgroup with lateral involvement, the distribution was as follows: KD IIIL in 16/29 cases (55.2%), KD IV in 7/29 (24.1%), KD V in 4/29 (13.8%) and KD IIIM in 2/29 (6.9%). Both IIIM cases displayed either a popliteus‐tendon rupture or iliotibial band rupture.

A PTFJ dislocation or subluxation was identified in 10 of 40 KDs ≥ III Schenck (Table 1). Eight of 10 KDs ≥ III received a specific treatment of the lateral ligament complex, in three of eight cases with Larson reconstruction. In two of 10 KDs ≥ III Schenck with PTFJ dislocation or subluxation no PLC injury was detected (patient nos. 4 and 5, Table 1). Lateral stress testing was negative in 6/10 KDs with PTFJ injury after either no PLC intervention, operative PLC reinsertion, or conservative management. As summarised in Table 1, varus stress testing revealed persistent instability of the lateral ligament complex in 3 of 10 knees with PTFJ dislocation or subluxation in KD ≥ III. In two of these three knees the PLC had been reconstructed using the Larson technique, while one knee had undergone reinsertion alone. Clinical follow‐up was unavailable for one knee with PTFJ subluxation (patient no. 9, Table 1).

There was no PTFJ injury in the ACL‐I group. The comparison of knee dislocation cohort and ACL‐insufficiency cohort with Mann–Whitney *U* test showed a significant difference in Fibula Lateralisation (*r* = 0.36; *p* = 0.001, Table 2). Proximal tibiofibular joints in the knee dislocation group also tended to have higher Inclination‐Horizontal compared to the PTFJs in the ACL‐insufficiency group (*r* = 0.22; *p* = 0.05).

**Table 2:** Comparison – knee dislocation (LUX) cohort vs. chronic anterior cruciate ligament insufficiency (ACL‐I) cohort.

	LUX (*n* = 40)			ACL‐I (*n* = 43)			
	Median	Min–Max	IQR	Median	Min–Max	IQR	*p* value
Age	49	21–85	27	28	17–55	18	< 0.001***
BMI (kg/m^2^)	28.3	20.0–53.3	7.3	25.7	18.5–44.4	7.2	0.109
Incl.‐hor. (°)	34.7	14.8–54.1	11.8	28.8	10.4–50.5	13.0	0.050
Incl.‐fib.axis (°)	65.1	50.8–84.5	12.8	65.9	34.8–82.5	13.1	0.913
Obliquity (°)	17.2	−15.7 to 46.1	16.6	20.1	−4.4 to 43.8	18.4	0.194
PFA (%)	94.8	18.5–100	15.9	100	57.0–100	18.0	0.528
FL (%)	0.0	−13.5 to 150.5	32.4	0.0	0.0–19.9	0.0	0.001**

*Note*: Mann–Whitney test.

Abbreviations: BMI, body mass index; Fib., fibula; FL, fibula lateralisation; Hor., horizontal; Incl., inclination; PFA, posterior fibula area; Post., posterior.

** *p* < 0.01

*** *p* < 0.001.

### Contralateral knee (CL) cohort

The contralateral cohort included 24 patients, 16 males (66.7%) and 8 females (33.3%), with a mean age of 53.5 years [26–85; 20] and a BMI of 28.8 kg/m² [20.5–41.5; 6.4]. The highest fibula lateralisation value observed in this group was 24.4%, and the lowest posterior fibula area value was 65.3%. A proximally medialized fibula (fibula lateralisation = ‐9.9%) was identified in one case (4.2%).

Contralateral knee cohort knees and matched ipsilateral knees did not differ with respect to the parameters inclination‐horizontal, inclination‐fibular axis, obliquity or posterior fibula area (Table 3). No PTFJ dislocation or subluxation was detected in contralateral cohort – whereas the matched knee dislocation group showed PTFJ subluxation in 9/24 cases (37,5%). In 88.9% (16/18) contralateral knee cohort knees matched that oft he ipsilateral side in terms of PTFJ type, a difference was observed in two patients (11.1%). The total knee dislocation cohort differed significantly from the contralateral knees in terms of fibula lateralisation (*r* = 0.29; *p* = 0.029, *n* = *64*), this difference remained when only matched contralateral knees were considered (r = 0.43; *p* = 0.007, *n* = 48, Table 3). No further differences in all examined variables were detected compared to the knee dislocation group (*p* ≥ 0.082).

**Table 3:** Comparison – knee dislocation (LUX) cohort vs. contralateral knee (CL) cohort.

	LUX (*n* = 24)			CL (*n* = 24)			
	Median	Min–Max	IQR	Median	Min–Max	IQR	*p* value
Age	53.5	26–85	20	53.5	26–85	20	1
BMI (kg/m^2^)	28.8	20.5–41.5	6.4	28.8	20.5–41.5	6.4	1
Incl.‐hor. (°)	34.9	18.7–54.1	12.2	36.1	20.7–49.0	10.4	0.620
Incl.‐fib.axis (°)	63.8	51.9–79.8	15.3	58.1	49.6–77.8	10.7	0.082
Obliquity (°)	17.9	−15.7 to 30.7	13.6	17.2	4.4–35.8	17.7	0.602
PFA (%)	92.5	63.5–100	15.0	86.2	65.3–100.0	22.2	0.208
FL (%)	16.1	−13.5 to 49.4	34.2	0.0	−9.9 to 24.4	1.2	0.007**

*Note*: Mann–Whitney test.

Abbreviations; BMI, body mass index; CL, contralateral knee cohort; FL, fibula lateralisation; LUX, knee dislocation cohort; PFA, posterior fibula area.

** *p* < 0.01.

**Figure 5:**
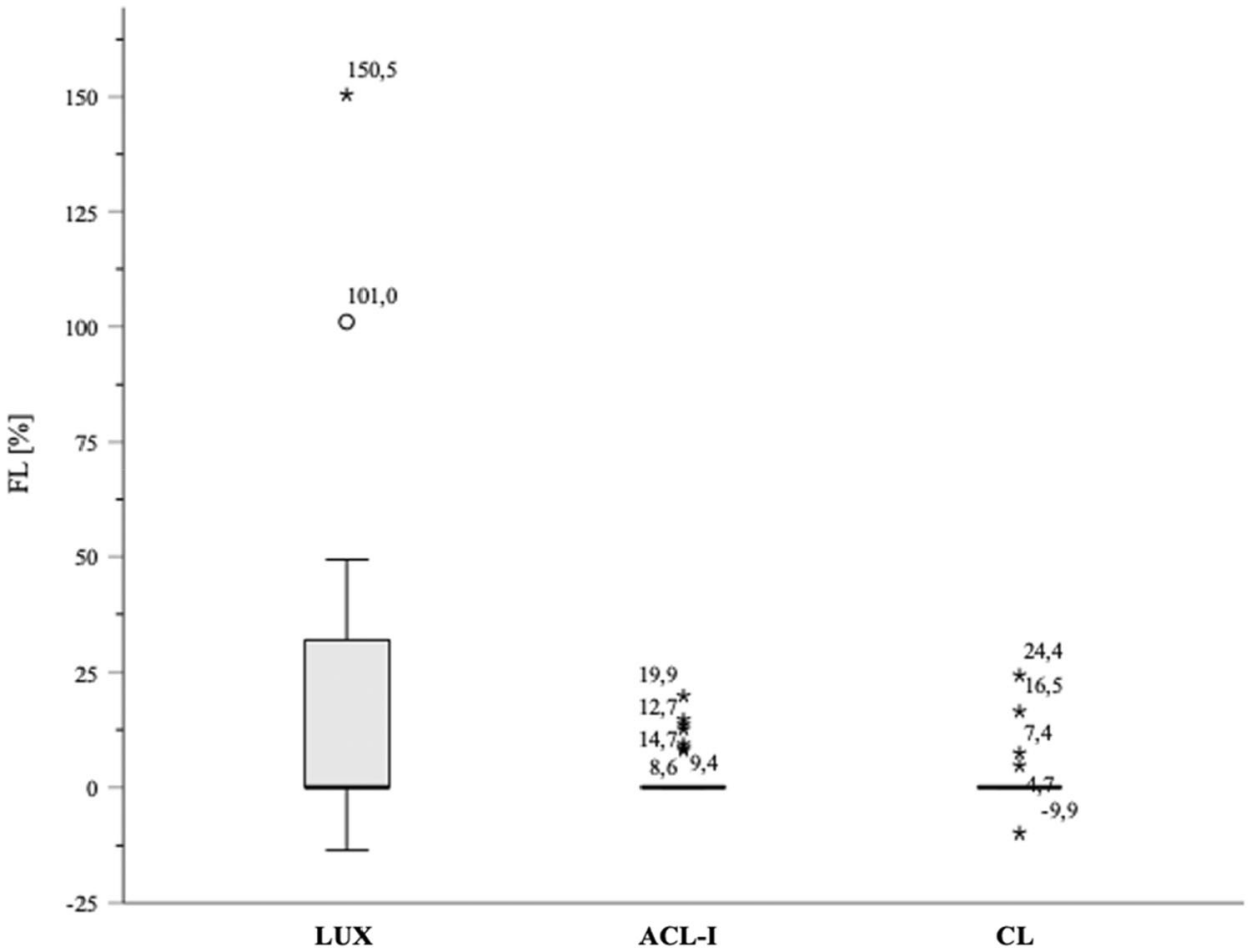
Boxplot – fibula lateralisation (FL). *Note*: ° = outliers (values more than 1.5× the interquartile range below the first quartile or above the third quartile), * = extreme outliers (values more than 3.0× the interquartile range below the first quartile or above the third quartile). ACL‐I, chronic anterior cruciate ligament insufficiency cohort; CL, contralateral knee cohort; LUX, knee dislocation cohort.

Figure 5 illustrates the fibula lateralisation differences among the subgroups, with complete dislocations represented as outliers in the knee dislocation cohort. Even extreme contralateral knee and ACL‐insufficiency group outliers do not exceed the third quartile of the knee dislocation cohort.

The intra‐rater agreement reached an intra‐class correlation coefficient (ICC) of 0.98 (CI 0.97–0.99; *p* < 0.001) for inclination‐horizontal, 0.85 (CI 0.72–0.92; *p* < 0.001) for inclination‐fibular axis, 0.90 (CI 0.80–0.95; *p* < 0.001) for PTFJ obliquity, 0.94 (CI 0.85–0.97; *p* < 0.001) for posterior fibula area, and 0.95 (CI 0.87–0.98; *p* < 0.001) for fibula lateralisation.

The inter‐rater agreement reached an ICC of 0.97 (CI 0.94–0.98; *p* < 0.001) for inclination‐horizontal, 0.93 (CI 0.78–0.97; *p* < 0.001) for inclination‐fibular axis, 0.89 (CI 0.79–0.94; *p* < 0.001) for PTFJ obliquity, 0.94 (CI 0.86–0.98; *p* < 0.001) for posterior fibula area and 0.96 (CI 0.90–0.98; *p* < 0.001) for fibula lateralisation.

## DISCUSSION

The most important finding of this study is the low prevalence of complete PTFJ dislocations in acute KDs classified as Schenck type ≥ III, with a rate of 5.4%, which is lower than previously reported in the literature. In contrast, the incidence of PTFJ dislocation in type IV KDs is high with 33.3%. The seldom‐discussed entity of PTFJ subluxation was indicated in 21.6% of the investigated KDs, following the definition above.

The diagnostic accuracy of PTFJ injuries remains insufficient to date, with even dominant anterolateral PTFJ dislocations being missed in approximately one‐third of cases [17]. Therefore, the aim of this study was to develop new parameters that allow detection of PTFJ dislocation and subluxations. The measurement methods fibula lateralisation and posterior fibula area demonstrated excellent intra‐ and inter‐rater agreement, making them suitable for qualitative and quantitative assessment of the PTFJ and its anterolateral dislocations. Their application is feasible using standard imaging software and does not impose high demands on the examiner.

Using the fibula lateralisation, the knee dislocation cohort could be clearly distinguished from ACL insufficiency and contralateral knee cohorts, as well as from matched contralateral sided PTFJs. Additionally, differences in fibula lateralisation were evident in joints classified as PTFJ dislocation or subluxation compared to other knees within the knee dislocation and the total cohort. The fibula lateralisation thus appears to be a reliable parameter for describing the coronal plane displacement of the proximal fibula relative to the tibial articular surface, both in healthy and injured knees.

Eight out of 10 PTFJs classified as dislocated or subluxated showed a posterior fibula area above the reference value of 63.5%. Only severe PTFJ dislocations with massive capsuloligamentous disruption exhibited an additional anterior fibular shift along with lateralisation [45], whereas in milder cases, the residual stabilising strength of the intact ligament complex appears to be sufficient to enable at least a.p. repositioning. This remaining function of the PTFJ ligaments, especially the APTFL, may explain the difficulty in diagnosing acute PTFJ subluxations or chronic PTFJ instability. It also suggests that fibula lateralisation is a more sensitive parameter compared to posterior fibula area for detecting PTFJ dislocations. The data from this study revealed eight PTFJ subluxations with a fibula lateralisation range between 27.7% and 49.4%. Unfortunately, specific clinical examination findings of the PTFJ are not available. However, it can be assumed that PTFJ subluxations may present with varying degrees of patient reported or clinical examined instability. The radiologically diagnosed subluxation may range from a clinically inapparent joint to one with marked instability. A normal curve of distribution ca be expected for the distribution of clinical manifestation.

Low posterior fibula area values should only be considered indicative of anterior displacement of the fibula after PTFJ obliquity has been assessed. A relationship between PTFJ obliquity and fibular positioning in the sagittal plane has already been described [41]. As shown by our data, a high obliquity is physiologically associated with a more anteriorly positioned fibula resulting in lower posterior fibula area values, as evidenced by the negative correlation between obliquity and posterior fibula area. Thus, a low posterior fibula area value may indicate an anterior dislocation of the proximal fibula only if PTFJ obliquity is low. Since factors such as age or meniscal configuration [40] may influence PTFJ morphology, larger datasets are needed to establish physiological reference values for the applied CT parameters in specific subgroups.

In 5.4% of patients with acute KD Schenck ≥ III a complete PTFJ dislocation was present, which is lower than in similar studies, reporting 9% in acute [16] and 10.7% in chronic KDs [33]. These differences are most likely attributable to variations in cohort characteristics and diagnostic criteria. Both studies [16, 33] used the Ballottement test in pre‐ or intraoperative anaesthesia examinations to evaluate PTFJ instability. However, the reliability of this methodology is limited, as side‐to‐side differences are not necessarily pathologic and the confounding factors of clinical examination methods are well known [5]. This approach appears to overestimate the prevalence of PTFJ instability in lower Schenck‐grade KDs, compared to radiological criteria in KDs classified as Schenck ≥ III. The CT based criteria defined in our study provide a stricter, more objective and reproducible assessment. However, it must be considered that dynamic examinations (e.g., clinical tests) produce different results than static assessments (e.g., supine CT scans), making a direct comparison between clinical and radiological stability tests difficult based on current data. Furthermore, identifying an unstable PTFJ does not necessarily indicate isolated insufficiency of PTFJ ligaments (APTFL and PPTFL), as besides these structures’ additional stabilisers such as the lateral collateral ligament [27] and interosseous membrane [1] play a crucial role. Additionally, the cohorts in previous mentioned studies were more heterogeneous, including patients with less severe injuries. Among the 213 patients included in the studies, 21 had PTFJ dislocations. Of these, 17 were classified as Schenck ≤ II, including 12 at stage I and two with mono‐ligament injuries accompanied by a popliteus tendon injury.

The ACL‐insufficiency cohort corresponds to chronic KDs classified as Schenck type I. We demonstrated that this cohort had a 0% prevalence of PTFJ injuries, significantly lower than in acute KDs classified as Schenck ≥ III. Therefore, the PTFJ appears to play a minor role in isolated chronic ACL injuries. In contrast PCL injuries frequently occur in combination with PLC injuries [11, 35], future research should thus focus on PTFJ in PCL involved entities.

As demonstrated by the subgroup of knee dislocations with PTFJ dislocation or subluxation, type IIIM dislocations may accompany with biceps femoris tendon injuries (1/3 KD IIIM). Another IIIM dislocation without PTFJ dislocation was found with popliteus tendon rupture. Thus, if a knee joint is described as IIIM dislocation a possibility remains, that an injury to the (postero‐) lateral capsuloligamentous structures is present. While PTFJ dislocation would primarily be anticipated in type IIIL KDs due to the posterolateral location of injury pattern, this may explain why PTFJ dislocations or subluxations cannot be excluded in type IIIM cases. This results from the Schenck classification not accounting for structures such as the biceps femoris tendon, the iliotibial band, or the popliteofibular complex. Particular attention should be paid to type IV KDs, as 33.3% of these cases involved complete PTFJ dislocation, which is a high incidence.

All 10 dislocated or subluxated PTFJs, as well as all fibular fractures, occurred in oblique‐type PTFJs. There was no conclusive difference between the knee dislocation group and the contralateral knee group with regard to the inclination fibular axis, as statistical confirmation was lacking in the matched contralateral knee group (p = 0.082). Oblique‐type PTFJs appear to be more susceptible to fractures and dislocations than horizontal‐type PTFJs, which is attributed to reduced external rotational capacity of oblique‐type PTFJs [27, 29].

This study is the first to analyse ipsilateral and matched contralateral PTFJs to assess whether both joints of an individual share similar anatomical characteristics. Among the investigated parameters, only the variable fibula lateralisation showed side‐to‐side differences, while no differences were found for the remaining variables. Furthermore, 88.9% of PTFJs exhibited concordant PTFJ types on both sides. These findings support the assumption of bilateral anatomical PTFJ similarity. However, since given that the knee dislocation cohort included seven subluxated PTFJs and additional joints with PLC injuries, the observed differences in FL were not unexpected.

The variables Inclination‐Horizontal, Inclination‐Fibular Axis and Obliquity are expected to remain unaffected by KD or PTFJ ligament injury. In contrast, a PTFJ injury or proximal fibular fracture may influence the parameters posterior fibula area and fibula lateralisation, making the knee dislocation cohort and their matched contralateral knees suitable only for comparison of inclination‐horizontal, inclination‐fibular axis and obliquity parameters. proper bilateral comparison of posterior fibula area and fibula lateralisation, would require a cohort with bilaterally uninjured PTFJs. Our results therefore suggest that the variables inclination‐horizontal, inclination‐fibular axis, and obliquity do not differ bilaterally in individuals.

### Strengths and limitations

An initial sample size calculation and a priori analysis was not feasible due to the lack of similar studies. The findings of this study are limited by the sample size, which is caused by the low prevalence of KDs and, more specifically, PTFJ dislocations and subluxations [26, 27]. Despite the demanding inclusion criteria (e.g., KD ≥ III), a solid cohort size could be achieved, comparable datasets for PTFJ analysis are currently not available in the existing literature. The Schenck classification is limited in its ability to fully capture the extent of injuries, as it only accounts for the ACL, PCL, lateral collateral ligament, medial collateral ligament, and fractures. This may result in underestimation of injuries involving, for example, the biceps femoris tendon, iliotibial band or the popliteofibular complex (e.g., patient no. 8).

For the first time, the above‐mentioned PTFJ‐specific measurement methods – previous mostly utilised on a standalone basis – were used in combination. The application of these parameters to dislocated PTFJs is also novel, as they have so far primarily been applied in specimens or healthy knee joints. The measurement methods used require minimal technical demands from the radiological software and are therefore broadly accessible.

The definition of PTFJ subluxations used in this study requires correlation to clinical assessment results of the PTFJ in future studies since, unlike a complete PTFJ dislocation, no reliable inference regarding the extent of clinical instability of PTFJ can be drawn from this parameter. Furthermore, no robust comparable data on PTFJ subluxations exist – apart from single case reports or small case series – which would allow our findings to be contextualised. That is why conclusions based on PTFJ subluxation data were formulated only with utmost restraint.

Due to the monocentric study design, a selection bias cannot be excluded; however, since the measurement methods demonstrated reliable results, a multicentric study design appears feasible for future investigations. The comparison between these contralateral and ipsilateral knees is further limited by the fact that the groups differ in terms of trauma and injuries to the posterolateral corner. Therefore, a potential selection bias related to the inclusion criteria of KD cannot be ruled out. An analysis of individuals with bilaterally uninjured knees would neutralise this limitation.

## CONCLUSION

This study demonstrates a 5.4% prevalence of PTFJ dislocation in knee dislocations ≥III, which is lower than previously reported. The incidence of PTFJ dislocation in knee dislocations type IV is high at 33.3%. Fibula lateralisation and posterior fibula area are suitable parameters for assessing anterolateral dislocation of the proximal fibula. Inclination‐horizontal, inclination‐fibular axis, and obliquity show no relevant side‐to‐side differences in individuals.

## AUTHOR CONTRIBUTIONS

Ben Louis Wagener was responsible for methodology, conceptualisation, investigation, formal analysis, statistical analysis, data curation, visualisations and writing the original draft. Timo Stausberg for investigation, statistical analysis and data curation. Thomas Rudolf Pfeiffer and Thomas Stein for conceptualisation, term, review and editing. Daniel Guenther for methodology, statistical analysis, conceptualisation, term, review, editing and supervision.

## CONFLICT OF INTEREST STATEMENT

The authors declare no conflicts of interest.

## ETHICS STATEMENT

The study was approved by the ethics committee of Witten‐Herdecke University (No. S‐01/2022) and followed the principles of the Declaration of Helsinki. All authors have read and approved the manuscript.

## Data Availability

De‐identified data can be provided on reasonable request.
